# Covid-19 impacts on unemployment on the migration-gender intersections in Spain, a multilevel study

**DOI:** 10.1093/eurpub/ckac129.315

**Published:** 2022-10-25

**Authors:** N Pedrós Barnils

**Affiliations:** Prevention and Health Promotion, Bremen University, Bremen, Germany

## Abstract

**Background:**

The covid-19 pandemic has led to several socioeconomic consequences, which ultimately affect individuals’ health status. This study aims to assess and explore the short-term impacts of the pandemic on the labour market in Spain, through the intersectional and multilevel analysis of unemployment rates.

**Methods:**

Cross-sectional study using data from the Spanish Labour Force Survey from the three quarters before and after the outbreak of covid-19 (N = 922,074). Multilevel logistic regressions were used to calculate the odds of being unemployed for the intersectional positions on gender and migration background, nested within 7 educational levels.

**Results:**

Before (pre) and after (post) the covid-19 outbreak, women had higher odds (ORnw-pre=1.974; p-value<0.001; ORmw-pre=2.202; p-value<0.001) of being unemployed than men (ORmm-pre= 0.887; p-value<0.001). However, the pandemic affected women's employment unequally. Migrant women experienced an increase in the risk of being unemployed 6 times larger than native women (48.67% (ORmw-post=2.688; p-value<0.001) v 8.78% (ORnw-post=2.062; p-value<0.001)). Moreover, in the post-covid 19 scenario, individual characteristics of migrant women (ORmw-post=2.688; p-value<0.001) played a larger role in the risk of being unemployed than their educational level (MOR=2.537).

**Conclusions:**

Migrant women are disproportionately bearing the short-term economic consequences of the pandemic by means of higher unemployment rates, underpinning experiences of racism and gender discrimination. Increasingly attention needs to be placed on discriminated groups in society. Targeted protective policies that foster labour market integration and increase social protection are needed to mitigate and decrease existing economic, social, and health inequalities.

**Key messages:**

• Migrant women have disproportionately carried the economic impact of the covid-19 pandemic in Spain. Targeted protective policies are needed to mitigate existing socioeconomic and health inequalities.

• Migrant women’s gender and migration background play a greater role than their education in the risk of being unemployed, underpinning experiences of racism and gender discrimination.

